# Editorial: Nutritional and physical activity strategies to boost immunity, antioxidant status and health, volume IV

**DOI:** 10.3389/fphys.2024.1379247

**Published:** 2024-02-16

**Authors:** Mallikarjuna Korivi, Arifullah Mohammed, Lukas Cipryan, Weibing Ye, Veeranjaneya Reddy Lebaka

**Affiliations:** ^1^ Institute of Human Movement and Sports Engineering, College of Physical Education and Health Sciences, Zhejiang Normal University, Jinhua, China; ^2^ Department of Agriculture Science, Faculty of Agro-Based Industry, Universiti Malaysia Kelantan, Jeli, Kelantan, Malaysia; ^3^ Department of Human Movement Studies, University of Ostrava, Ostrava, Czechia; ^4^ Department of Microbiology, Yogi Vemana University, Kadapa, India

**Keywords:** physical activity, caffeine, prediabetes, mental health, Yijinjing exercise

Physical activity and dietary habits play a fundamental role in maintaining and promoting of physical fitness and health in all age group of people. Evidence from our previous Research Topic collections (Volume I, II and III) demonstrated that nutritional intervention or exercise training alone or in combination of both promotes overall health by boosting antioxidant, inflammation and/or immune systems in animals and humans. In clinical context, regular exercise training with or without nutritional intervention has been shown to improve various clinical outcomes in patients with diabetes, cardiovascular disease, depression, liver disease and metabolic syndrome (Korivi et al., 2022a; Korivi et al., 2022b; Korivi et al., 2023). In the present Research Topic collection (Volume IV), we continued to emphasize the beneficial effects of physical activity and nutrition, particularly on endurance performance, muscle physiology and overall wellbeing. This volume consisted of a six interesting articles, including four original research findings, one clinical trial and one systematic review.

A double-blind crossover study by Wang and colleagues demonstrated that low dose of caffeinated energy drink supplementation improved exercise performance in young male triathletes, while high dose of caffeinated energy drink deceased performance (Wang et al.). Noteworthy, either low or high dose of caffeinated energy drink supplementation had no effect on cortisol or testosterone levels, but decreased lipid peroxidation in triathletes (Wang et al.). Recent reports are suggesting that exercising location or environment is a key player in promoting the mood or physical fitness of individuals. A study conducted to evaluate the influence of exercising environment on changes in blood pressure, heart rate variability and positive/negative affects among adults. Findings showed that exercising in green space and horticultural activity with plants, both showed a similar effect on decreasing blood pressure. However, horticultural activity with plants appears to be better in decreasing the negative affects among participants compared to green exercise (Tao et al.). These findings suggest that either green exercise or horticultural activities can promote the physiological parameters, but horticultural activity might be suitable for psychological improvements.

Regarding the exercise type, Tai Chi, Yijinjing, Qigong and square dance are the conventional exercise patterns in Chinese society, which can be performed by controlling the body movements. In our 2nd volume, we explored the beneficial effects of Tai Chi and square dance on immune function, physical health and life satisfaction in older adults (Korivi et al., 2022b). In this 4th volume, another study disclosed the beneficial effects of traditional Chinese mind-body exercise known as “Yijinjing” in middle-aged and elderly adults diagnosed with prediabetes (Huang et al.). In this clinical trial, Yijinjing combined with elastic band exercise significantly decreased bodyweight, body mass index, leg fat mass and total body fat mass, while delayed loss of lean mass in patients. In addition, practicing of Yijinjing with band exercise for 6-month contributed to decrease fasting blood glucose, 2-h postprandial blood glucose, insulin, insulin resistance index and total cholesterol. Other clinical measures, such as growth hormone and 25-hydroxyvitamin D were gradually increased following Yijinjing and elastic band exercise in patients with prediabetes (Huang et al.).

In sports, the dynamic balance ability is important to achieve highest competitive performance and to avoid incidence of lower-body musculoskeletal injuries, such as knee and ankle sprains. A study by Huang et al. evaluated whether lower quarter y-balance test (YBT-LQ) could be a reliable predictor of sports injury risk among Chinese physical education college students. The findings showed a strong correlation of YBT-LQ with sports performance and injury, while moderate correlation with physical activity, metabolic equivalent and age (negative) of participants (Huang et al.). This study provides valuable insights into how strategic exercise serves as a tool for maintaining health and minimizing risk of injury in sports.

On the other hand, massage is widely used as therapy to decrease muscle stiffness, increase joint range of motion and prevent delayed onset of muscle soreness, which together can promote exercise performance. A study by Qu and colleagues Qu et al. demonstrated the beneficial effects of massage among adolescent wrestlers. In this study, 2-week armchair machine massage reported to decrease the dynamic stiffness of erector spinae as well as serum creatine kinase levels in wrestlers. These findings imply that machine massage is able to improve physical properties of muscles (reduce stiffness and increase elasticity), and thereby promote athletic performance among adolescent wrestlers (Qu et al.). In adolescents, physical activity is crucial not only for physical fitness but also for psychological wellbeing. In a systematic review, Shao and Zhou emphasized the importance of physical activity among adolescents irrespective of gender, age and ethnicity. Briefly, adolescents with less physical activity had high body mass index, whereas adolescents with high self-efficacy and satisfaction tend to have high physical activity behavior. In addition, negative life style factors, including sedentary behavior, smoking, excessive screen time and negative emotions were correlated with decreased physical activity habit in adolescents. Encouragement from parents, teachers and friends, and bringing awareness of physical activity benefits could help adolescents in engage physical activity programs (Shao and Zhou).

This Research Topic collection concludes that exercise in any form at any location is beneficial to promote physical and psychological health in all age groups of people with or without diseases. Therefore, it is highly recommended to engage in regular exercise programs with proper dietary patterns to treat and/or prevent physiological disorders in chronic diseases.
